# TXNDC12 inhibits lipid peroxidation and ferroptosis

**DOI:** 10.1016/j.isci.2023.108393

**Published:** 2023-11-04

**Authors:** Lanlan Tang, Yan Yu, Wenjun Deng, Jiao Liu, Yichun Wang, Fanghua Ye, Rui Kang, Daolin Tang, Qingnan He

**Affiliations:** 1 Department of Pediatrics, The Third Xiangya Hospital, Central South University, Changsha, Hunan 410013, China; 2 Department of Pediatrics, Xiangya Hospital, Central South University, Changsha, Hunan 410008, China; 3 DAMP Laboratory, The Third Affiliated Hospital of Guangzhou Medical University, Guangzhou, Guangdong 510150, China; 4 Department of Critical Care Medicine, The Third Affiliated Hospital of Guangzhou Medical University, Guangzhou, Guangdong 510150, China; 5 Department of Surgery, UT Southwestern Medical Center, Dallas, TX 75390, USA

**Keywords:** Biological sciences, Biochemistry, Molecular biology, Cell biology

## Abstract

Ferroptosis is a type of regulated cell death characterized by lipid peroxidation and subsequent damage to the plasma membrane. Here, we report a ferroptosis resistance mechanism involving the upregulation of TXNDC12, a thioredoxin domain-containing protein located in the endoplasmic reticulum. The inducible expression of TXNDC12 during ferroptosis in leukemia cells is inhibited by the knockdown of the transcription factor ATF4, rather than NFE2L2. Mechanistically, TXNDC12 acts to inhibit lipid peroxidation without affecting iron accumulation during ferroptosis. When TXNDC12 is overexpressed, it restores the sensitivity of ATF4-knockdown cells to ferroptosis. Moreover, TXNDC12 plays a GPX4-independent role in inhibiting lipid peroxidation. The absence of TXNDC12 enhances the tumor-suppressive effects of ferroptosis induction in both cell culture and animal models. Collectively, these findings demonstrate an endoplasmic reticulum-based anti-ferroptosis pathway in cancer cells with potential translational applications.

## Highlights

• TXNDC12 is upregulated during ferroptosis
• ATF4 is required for TXNDC12 expression during ferroptosis
• TXNDC12 acts as a suppressor of ferroptosis by inhibiting lipid peroxidation
• Targeting TXNDC12 enhances ferroptosis-mediated tumor suppression *in vivo*

## Graphical abstract

**Figure:**
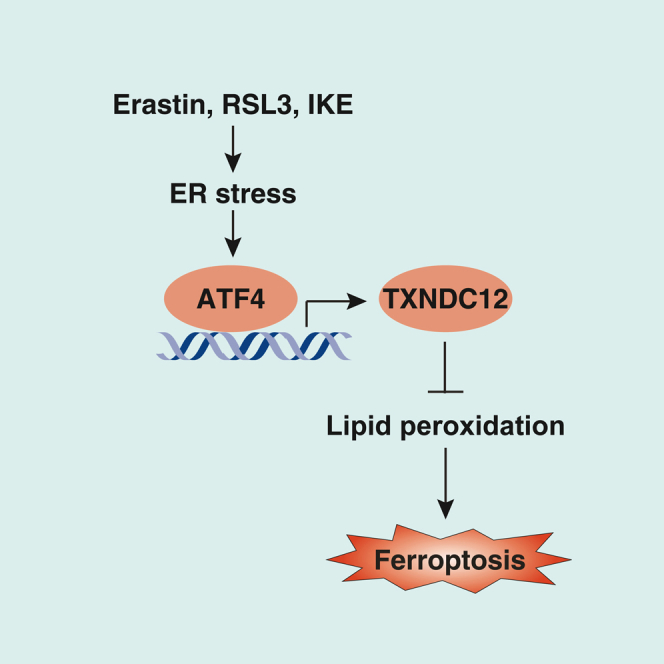

## Introduction

Chemotherapy is a widely utilized treatment for cancer that aims to kill cancer cells or inhibit their growth.^1^ Various types of chemotherapy drugs exert their effects through different mechanisms, including the induction of regulated cell death.^2^ Regulated cell death can occur through multiple forms, including apoptotic and non-apoptotic cell death.^3^ Traditionally, most chemotherapy agents are designed to induce caspase-dependent apoptosis through different molecular targets, aiming to inhibit the proliferation of tumor cells.^4^ For example, alkylating agents directly damage the DNA of cancer cells, preventing replication and leading to apoptotic cell death.^5^ Examples of such agents include cyclophosphamide, cisplatin, and temozolomide. However, cancer cells can develop mechanisms (e.g., autophagy) to evade apoptosis induced by chemotherapy, which can result in treatment resistance and disease progression.^6^^,^^7^^,^^8^ As an alternative approach to combat tumors, recent studies have emphasized the induction of non-apoptotic cell death. Among these, ferroptosis, an iron-dependent form of non-apoptotic cell death,^9^ has shown potent anticancer effects in chemotherapy-resistant cells.^10^^,^^11^^,^^12^^,^^13^^,^^14^

Multiple molecular and organelle pathways are involved in the regulation of ferroptosis induction and execution.^15^^,^^16^^,^^17^ One key anti-ferroptosis pathway is the system xc^−^-glutathione peroxidase 4 (GPX4) pathway.^9^^,^^18^^,^^19^ The system xc^−^ is a cystine and glutamate antiporter located on the cell membrane, composed of two subunits: solute carrier family 7 member 11 (SLC7A11; also known as xCT) and solute carrier family 3 member 2 (SLC3A2; also known as CD98). This antiporter is responsible for the exchange of extracellular cystine with intracellular glutamate. Cystine is reduced to cysteine, which acts as the limiting precursor for the synthesis of intracellular glutathione (GSH), a pivotal antioxidant molecule.^20^^,^^21^ The enzymatic activity of GPX4 relies on the presence of reduced GSH to carry out its function. GPX4 plays a crucial role in protecting cells from oxidative stress and lipid peroxidation by reducing lipid hydroperoxides into their corresponding alcohols.^22^

Classical ferroptosis inducers, such as erastin and RSL3, function as inhibitors of system xc^−^ and GPX4, respectively.^9^^,^^18^ While these findings provide strong evidence linking disrupted GPX4 pathways and ferroptosis induction, the modulation of ferroptosis can also occur through GPX4-independent mechanisms. For example, apoptosis inducing factor mitochondria associated 2 (AIFM2)^23^ and dihydroorotate dehydrogenase (DHODH)^24^ play a GPX4-independent role in preventing ferroptosis in the cell membrane and mitochondria, respectively. While multiple organelles can influence ferroptosis signaling, the endoplasmic reticulum (ER) appears to play a central role in mediating ferroptosis.^25^ However, the specific antioxidant pathways within the ER that regulate ferroptosis sensitivity remain poorly understood.

The thioredoxin domain-containing (TXNDC) family comprises 17 members within the protein disulfide isomerase (PDI) family. They play a pivotal role in numerous cellular homeostatic mechanisms, including the facilitation of proper protein folding through their disulfide isomerase activity and assistance in electron transfer to other oxidoreductases.^26^ Despite their significant antioxidative functions, the precise effects and underlying mechanisms by which TXNDC proteins defend against ferroptosis remain largely elusive.

In this study, we present evidences regarding the role of thioredoxin domain-containing protein 12 (TXNDC12; also known as ERp18 or ERp19), a protein predominantly situated in the ER of humans and other animals.^27^^,^^28^ We demonstrate that the expression of TXNDC12 is upregulated in human leukemia cells treated with erastin or RSL3, resulting in increased resistance to ferroptosis. Moreover, elevated baseline expression of TXNDC12 in human solid cancer cells (e.g., colorectal cancer cells) is also associated with resistance to ferroptosis. Conversely, genetic knockdown of *TXNDC12* enhances ferroptosis sensitivity by inducing lipid peroxidation. Targeting TXNDC12 also enhances the anticancer activity of ferroptosis-mediated tumor suppression *in vivo*. These findings not only unveil a key anti-ferroptotic pathway within the ER, but also suggest a promising therapeutic strategy for combating tumors by targeting the TXNDC12 pathway.

## Results

### TXNDC12 is upregulated during ferroptosis

**Figure 1:**
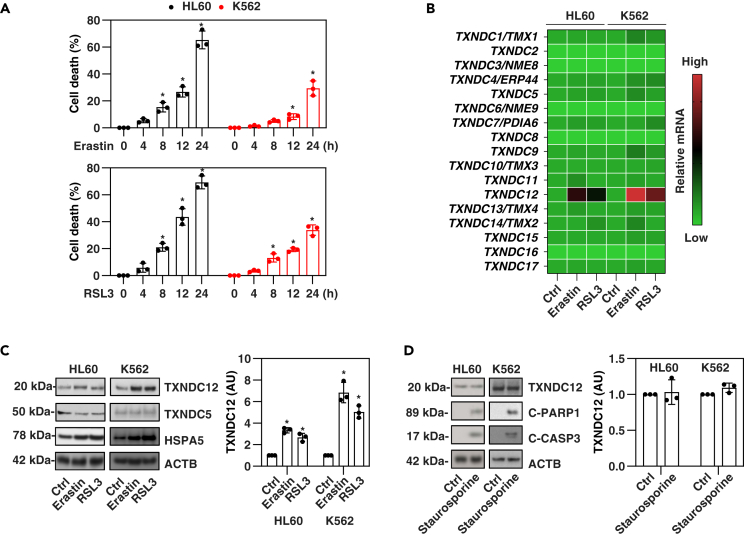
TXNDC12 is upregulated during ferroptosis (A) HL60 and K562 cells were treated with erastin (10 μM) or RSL3 (0.5 μM) for 4–24 h, and cell death was assessed. The data represent the means ± SD from three independent samples. Statistical analysis was performed using two-way ANOVA with Tukey’s multiple comparisons test. ∗p < 0.05 versus control group. (B) HL60 and K562 cells were treated with erastin (10 μM) or RSL3 (0.5 μM) for 24 h, and the mRNA expression of *TXNDC* family genes was evaluated. The data represent a heatmap of means from three independent samples. (C, and D) Western blot analysis was conducted to assess the expression of indicated proteins in HL60 or K562 cells treated with erastin (10 μM), RSL3 (0.5 μM) (C), or staurosporine (0.5 μM) (D) for 24 h. The right panel illustrates the semi-quantitative expression of the TXNDC12 protein, with the control set as 1. Statistical analysis was performed using two-way ANOVA with Tukey’s multiple comparisons test. ∗p < 0.05 versus control group.

Ferroptosis is closely linked to ER stress.^29^^,^^30^^,^^31^ Among the members of the protein disulfide isomerase family, TXNDC12 plays a crucial role in defending against ER stress.^27^^,^^28^ To investigate the role of TXNDC12 in ferroptosis, we performed an initial assay to evaluate the expression of TXNDC12 in two human leukemia cell lines (HL60 and K562). Consistent with the previous study,^32^ HL60 cells were more sensitive than K562 cells to erastin or RSL3 (Figure 1A). Quantitative PCR (qPCR) analysis demonstrated a significant upregulation of *TXNDC12* mRNA in response to erastin or RSL3, particularly observed in surviving K562 cells (Figure 1B). In contrast, other 16 members of the *TXNDC* gene family did not exhibit the same level of upregulation as *TXNDC12* mRNA in HL60 and K562 cells (Figure 1B). Western blot analysis provided further confirmation that the protein expression of TXNDC12 (distinct from the extensively studied TXNDC family member, TXNDC5, in disease^33^) was upregulated leukemia cells, particularly in K562 cells, following treatment with erastin or RSL3 (Figure 1C). As a positive control, the expression of heat shock protein family A (Hsp70) member 5 (HSPA5), a known marker of ER stress associated with ferroptosis,^31^ was also upregulated (Figure 1C). Furthermore, the upregulation of TXNDC12 protein was not observed in K562 and HL60 cells undergoing apoptosis induced by staurosporine (Figure 1D). As a positive control, the presence of cleaved-caspase 3 (CASP3) and cleaved-poly(ADP-ribose) polymerase 1 (PARP1; a caspase substrate) was observed in response to staurosporine treatment (Figure 1D). These findings suggest that the upregulation of TXNDC12 is specific to ferroptosis stimulation, rather than an apoptosis activator.

### ATF4 is required for TXNDC12 expression during ferroptosis

**Figure 2:**
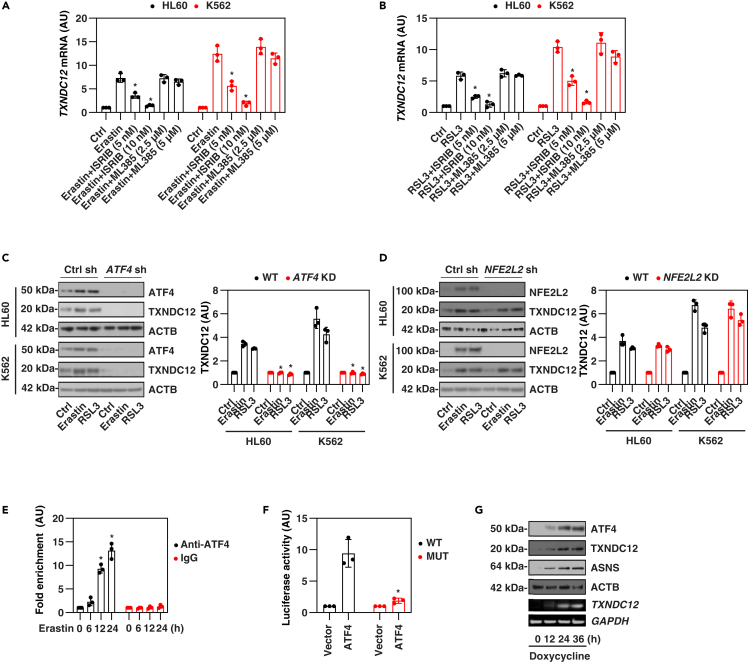
ATF4 is required for TXNDC12 upregulation during ferroptosis (A and B) HL60 and K562 cells were treated with erastin (10 μM) (A) or RSL3 (0.5 μM) (B) in the absence or presence of ISRIB (5 and 10 nM) or ML385 (2.5 and 5 μM) for 24 h, and the mRNA expression of *TXNDC12* was assessed. The data represent the means ± SD from three independent samples. Statistical analysis was performed using two-way ANOVA with Tukey’s multiple comparisons test. ∗p < 0.05 versus erastin or RSL3 group. (C and D) Wild-type, *ATF4*-KD (C) or *NFE2L2*-KD (D) leukemia cells were treated with erastin (10 μM) or RSL3 (0.5 μM) for 24 h, and the protein levels of TXNDC12 were assessed by western blot. The right panel illustrates the semi-quantitative expression of the TXNDC12 protein, with the control set as 1. Statistical analysis was performed using two-way ANOVA with Tukey’s multiple comparisons test. ∗p < 0.05 versus WT group. (E) ChIP analysis of putative ATF4 binding motif using anti-ATF4 antibodies or control IgG antibodies in K562 cells after erastin (10 μM) treatment for 6–24 h. The data represent the means ± SD from three independent samples. Statistical analysis was performed using two-way ANOVA with Tukey’s multiple comparisons test. ∗p < 0.05 versus IgG group. (F) Luciferase reporter assay in 293FT cells transfected with reporter containing WT *TXNDC12* promoter or ATF4 response element mutant *TXNDC12* promoter (MUT), together with vector or *ATF4* expression plasmids (pMIEG3-ATF4). Firefly luciferase activity was normalized to Renilla activity. The data represent the means ± SD from three independent samples. Statistical analysis was performed using two-way ANOVA with Tukey’s multiple comparisons test. ∗p < 0.05 versus WT group. (G) Western blot and RT-PCR for tet-on ATF4 cells treated with doxycycline.

To investigate the upregulation mechanism of TXNDC12 during ferroptosis, our focus was on two key transcription factors, activating transcription factor 4 (ATF4) and like BZIP transcription factor 2 (NFE2L2; also known as NRF2), known to be involved in ER stress and antioxidant response during ferroptosis.^31^^,^^34^^,^^35^^,^^36^^,^^37^^,^^38^ In HL60 and K562 cells, we observed that the ATF4 inhibitor ISRIB, rather than the NFE2L2 inhibitor ML385, effectively and dose-dependently suppressed the upregulation of *TXNDC12* mRNA induced by erastin or RSL3 (Figures 2A and 2B), indicating that ATF4 may mediate TXNDC12 expression during ferroptosis.

To further validate the requirement of ATF4 for TXNDC12 upregulation, we employed specific shRNA to suppress the expression of *NFE2L2* or *ATF4* in HL60 and K562 cells, respectively (Figures 2C and 2D). As expected, erastin or RSL3 treatment led to the upregulation of NFE2L2 and ATF4, both being stress-inducible transcription factors (Figures 2C and 2D). Nevertheless, the knockdown of *ATF4*, rather than *NFE2L2*, led to the inhibition of TXNDC12 protein expression in HL60 and K562 cells after exposure to erastin or RSL3 (Figures 2C and 2D).

Next, we conducted a bioinformatics analysis on the promoter sequence of the human *TXNDC12* gene, which revealed the presence of the putative ATF4 binding motif located between positions −584 to −571 base pairs (ATCAGTTGGTTGCA). Subsequent chromatin immunoprecipitation (ChIP) analysis confirmed the binding of ATF4 to this specific ATF4 response element following erastin treatment in K562 cells (Figure 2E). To investigate the impact of this ATF4 response element on *TXNDC12* promoter activity, we conducted transfection experiments in 293FT embryonic kidney cells. We introduced luciferase reporter constructs containing a 1000-bp segment of the *TXNDC12* promoter, as well as a variant of this promoter with a mutated ATF4 binding region. Concurrently, we transfected an *ATF4* expression plasmid into these cells. The results demonstrated an increase in luciferase activity in the presence of the ATF4 plasmid when using the wide type (WT) promoter reporter construct (Figure 2F). In contrast, the mutation of the ATF4 response element led to a reduction in luciferase activity in the *TXNDC12* promoter reporter (Figure 2F). Furthermore, we conducted an experiment using a tetracycline-controlled (tet-on) ATF4-inducible cell line, which subsequently confirmed that TXNDC12 is indeed a gene regulated by ATF4 (Figure 2G). As a positive control,^39^ the protein expression of *ATF4*-targeted gene asparagine synthetase (glutamine-hydrolyzing) (ASNS) was also upregulated when treated with doxycycline (Figure 2G).

Taken together, these findings suggest that the activation of the transcription factor ATF4 contributes to TXNDC12 expression during ferroptosis.

### TXNDC12 functions as a repressor of ferroptosis

**Figure 3:**
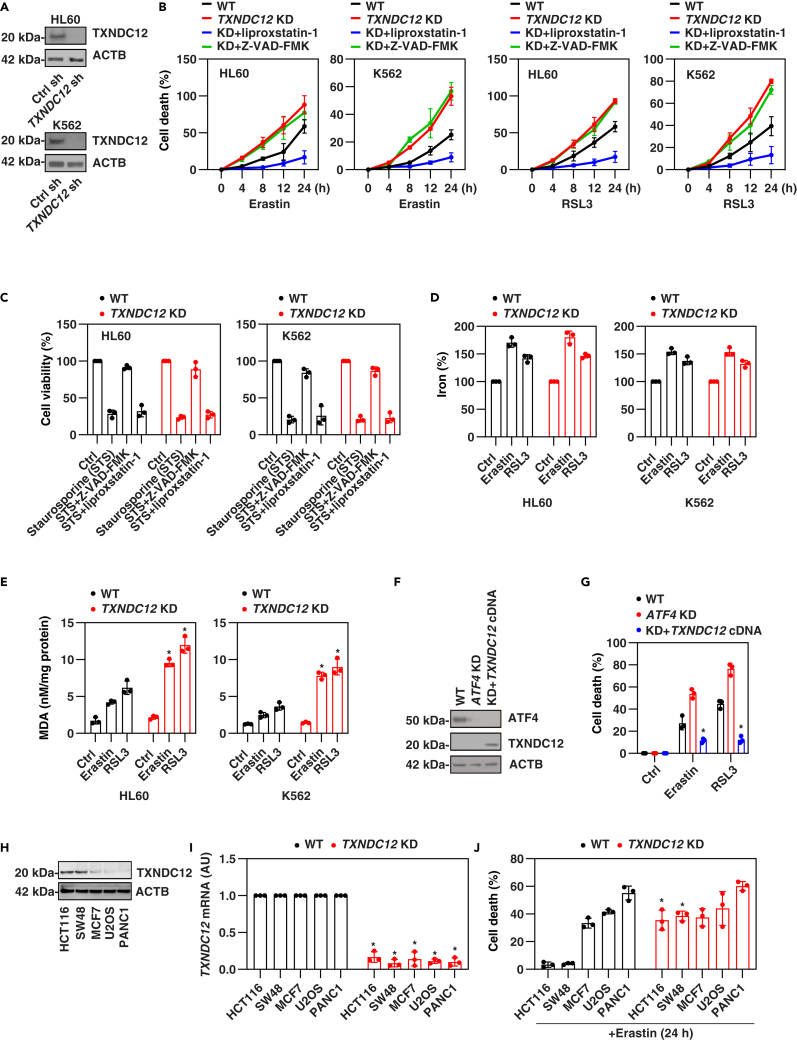
Knockdown of TXNDC12 increases ferroptosis (A) Protein expression of TXNDC12 was analyzed by western blot in indicated HL60 and K562 cells. (B) The indicated cells were treated with erastin (10 μM) or RSL3 (0.5 μM) for 24 h in the absence or presence of liproxstatin-1 (1 μM) or Z-VAD-FMK (10 μM), and cell death was assessed. The data represent the means ± SD from three independent samples. (C) The indicated cells were treated with staurosporine (0.5 μM) for 24 h in the absence or presence of liproxstatin-1 (1 μM) or Z-VAD-FMK (10 μM), and cell viability was assessed. The data represent the means ± SD from three independent samples. (D and E) The indicated cells were treated with erastin (10 μM) or RSL3 (0.5 μM) for 24 h, and intracellular iron (D) and MDA (E) were assessed. The data represent the means ± SD from three independent samples. Statistical analysis was performed using two-way ANOVA with Tukey’s multiple comparisons test. ∗p < 0.05 versus WT group. (F) Western blot analysis of protein expression in indicated K562 cells. (G) The indicated K562 cells were treated with erastin (10 μM) or RSL3 (0.5 μM) for 24 h, and cell death were assessed. The data represent the means ± SD from three independent samples. Statistical analysis was performed using two-way ANOVA with Tukey’s multiple comparisons test. ∗p < 0.05 versus *ATF4* KD group. (H) Western blot analysis of TXNDC12 expression in indicated cancer cell lines. (I) qPCR analysis of *TXNDC12* mRNA expression in indicated WT and *TXNDC12-*KD cells. The data represent the means ± SD from three independent samples. Statistical analysis was performed using two-way ANOVA with Tukey’s multiple comparisons test. ∗p < 0.05 versus WT group. (J) The indicated WT and *TXNDC12*-KD cancer cells were treated with erastin (5 μM) for 24 h and cell death were assessed. The data represent the means ± SD from three independent samples. Statistical analysis was performed using two-way ANOVA with Tukey’s multiple comparisons test. ∗p < 0.05 versus WT group.

To investigate the role of TXNDC12 in ferroptosis, we employed specific shRNA to suppress its expression in HL60 and K562 cells (Figure 3A). The depletion of *TXNDC12* resulted in a significant increase in cell death induced by erastin or RSL3 (Figure 3B). This effect was reversed by ferroptosis inhibitor liproxstatin-1 (Figure 3B). However, the apoptosis inhibitor Z-VAD-FMK (pan-caspase inhibitor) did not have effect on erastin- or RSL3-induced cell death in *TXNDC12*-knockdown HL60 and K562 cells (Figure 3B), although it inhibited staurosporine-induced growth inhibition (Figure 3C). In contrast, the knockdown of *TXNDC12* or the administration of liproxstatin-1 had no effect on staurosporine-induced growth inhibition in HL60 and K562 cells (Figure 3C). This suggests that TXNDC12 is not a key regulator of apoptosis sensitivity.

**Figure 4:**
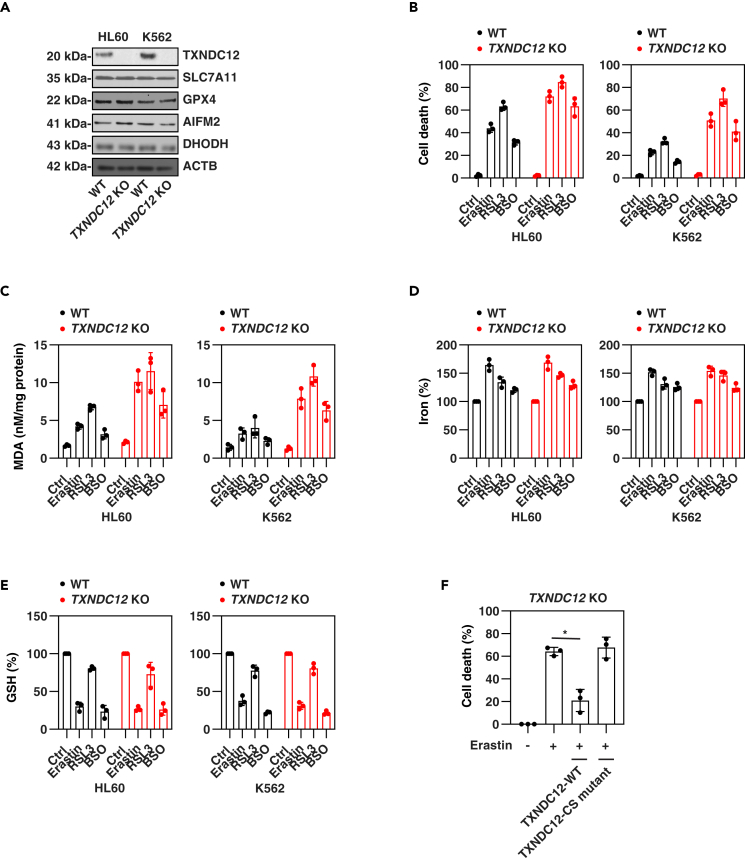
Knockout of TXNDC12 increases ferroptosis (A) Western blot analysis of indicated protein expression in WT and *TXNDC12*-knockout (KO) HL60 and K562 cells. (B–E) The indicated HL60 and K562 cells were treated with erastin (10 μM), RSL3 (0.5 μM) or BSO (100 μM) for 24 h, and cell death (B), intracellular MDA (C), iron (D), and GSH (E) were assessed. The data represent the means ± SD from three independent samples. (F) *TXNDC12*-KO K562 cells were subjected to transfection with either wild-type *TXNDC12* or a cysteine-mutated variant of *TXNDC12* (CS), followed by a 24-h treatment with erastin. Cell death was subsequently assessed. The data represent the means ± SD from three independent samples. Statistical analysis was performed using two-way ANOVA with Tukey’s multiple comparisons test. ∗p < 0.05.

Furthermore, we assessed key biochemical events of ferroptosis, including intracellular iron levels and lipid peroxidation.^16^^,^^40^^,^^41^ Consistent with previous studies,^9^ erastin exhibited a stronger activity in inducing iron accumulation compared to RSL3 (Figure 3D). However, our assays indicated that erastin- or RSL3-induced iron accumulation in HL60 and K562 cells during ferroptosis was unaffected by the knockdown of *TXNDC12* (Figure 3D), despite a recent study suggesting that TXNDC12 may inhibit iron accumulation in glioma cells.^42^ In contrast, the absence of *TXNDC12* accelerated erastin- or RSL3-induced lipid peroxidation, as evidenced by the malondialdehyde (MDA) assay (Figure 3E), which detects the highly mutagenic byproduct of lipid peroxidation, during ferroptotic stimulation. Furthermore, CRISPR-Cas9-mediated knockout of *TXNDC12* in HL60 or K562 cells (Figure 4A) resulted in an augmentation of erastin-, RSL3- or buthionine sulfoximine (BSO, an inhibitor of gamma-glutamylcysteine synthetase)-induced cell death (Figure 4B), accompanied by an increase in MDA production (Figure 4C). However, the knockout of *TXNDC12* had no significant impact on the levels of iron (Figure 4D) and GSH (Figure 4E) in the absence or presence of erastin, RSL3, or BSO treatment. The protein expression of key regulators of ferroptosis, including SLC7A11, GPX4, AIFM2, and DHODH, also remained unaffected by the *TXNDC12* knockout (Figure 4A).

To further investigate whether the enzymatic activity of PDI in TXNDC12 is necessary for its antiferrotitic function, we utilized the TXNDC12-CS mutant, in which both cysteines in the active site were mutated.^43^ The expression of WT TXNDC12, rather than the TXNDC12-CS mutant, reinstated the resistance of *TXNDC12*-knockout K562 cells to erastin (Figure 4F). This suggests that the active site of TXNDC12 plays a crucial role in TXNDC12-mediated antiferrotitic function. In addition, the overexpression of *TXNDC12* restored the resistance of *ATF4*-knockdown cells to erastin or RSL3 (Figures 3F and 3G). Therefore, the expression of TXNDC12, regulated by ATF4, may function as a negative regulator of lipid peroxidation and subsequent ferroptosis in leukemia cells.

To further investigate whether TXNDC12 expression is a general mechanism of ferroptosis resistance, we assessed the levels of TXNDC12 expression in erastin-sensitive cancer cell lines (MCF7, U2OS, and PANC1) and erastin-resistant cancer cell lines (HCT116 and SW48) based on previous research findings.^31^^,^^44^^,^^45^^,^^46^ HCT116 and SW48 exhibited higher levels of TXNDC12 expression when compared to MCF7, U2OS, and PANC1 cells (Figure 3H). Functionally, the *TXNDC12* knockdown showed a more effective reversal of erastin resistance in HCT116 and SW48 cells compared to MCF7, U2OS, and PANC1 cells (Figures 3I and 3J).

### TXNDC12 inhibits lipid peroxidation

**Figure 5:**
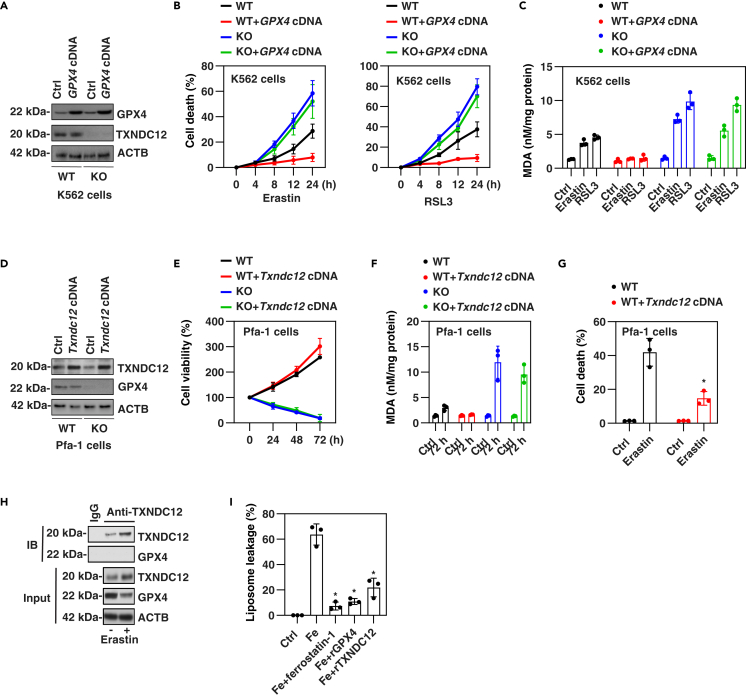
TXNDC12 inhibits ferroptosis independent on GPX4 (A) The protein expression of GPX4 was analyzed by western blot in indicated K562 cells with or without *GPX4* overexpression. (B) Cell death analysis was performed on the indicated K562 cells with or without *GPX4* overexpression in response to erastin (10 μM) or RSL3 (0.5 μM) for 4–24 h. (C) The levels of MDA were analyzed in the indicated K562 cells with or without *GPX4* overexpression in response to erastin (10 μM) or RSL3 (0.5 μM) for 24 h. (D) The protein expression of TXNDC12 was analyzed by western blot in indicated WT and *Gpx4*-knockout Pfa-1 cells with or without *Txndc12* overexpression. (E) Cell death analysis was performed on the indicated Pfa-1 cells with or without *Txndc12* overexpression. (F) The levels of MDA were analyzed in the indicated Pfa-1 cells at 72 h. (G) Cell death analysis was performed on the indicated Pfa-1 cells in response to erastin (5 μM) for 24 h. The data represent the means ± SD from three independent samples. Statistical analysis was performed using two-way ANOVA with Tukey’s multiple comparisons test. ∗p < 0.05 versus WT group. (H) IP analysis was performed on K562 cells in response to erastin (10 μM) for 12 h. (I) Fluorescent calcein dye was entrapped within liposomes, which were subsequently exposed to Fe^2+^ (10 μM) under conditions either with or without the presence of ferrostain-1 (1 μM), rGPX4 (200 nM), or rTXNDC1 (200 nM) for a duration of 20 min. The release of calcein from the liposomes was assessed by measuring changes in calcein fluorescence intensity. The data represent the means ± SD from three independent samples. Statistical analysis was performed using one-way ANOVA with Tukey’s multiple comparisons test. ∗p < 0.05 versus Fe group.

GPX4 plays a crucial role in maintaining oxidative homeostasis and defending against oxidative stress during ferroptosis.^18^^,^^47^^,^^48^ However, besides GPX4, there are also GPX4-independent antioxidant pathways that contribute to the regulation of ferroptotic damage.^49^ To determine whether the inhibitory effect of TXNDC12 on ferroptosis is dependent on GPX4, we conducted overexpression experiments of *GPX4* in WT and *TXNDC12*-knockout K562 cells. After gene transfection of *GPX4* cDNA, the protein expression of GPX4 increased similarly in both WT and *TXNDC12*-knockout K562 cells (Figure 5A). The cell death assay suggested that elevating GPX4 levels impeded the anticancer activity of erastin or RSL3 in WT cells, rather than in *TXNDC12*-knockout K562 cells (Figure 5B). Furthermore, the MDA assay indicated that the overexpression of *GPX4* was unable to reverse lipid peroxidation in *TXNDC12*-knockout K562 cells compared to WT cells (Figure 5C). This suggests that the deficiency of *TXNDC12* increased lipid peroxidation and the sensitivity of K562 cells to ferroptosis.

Notably, despite the overexpression of *Txndc12*, cell viability and lipid peroxidation remained unaltered in inducible *Gpx4*-deficient Pfa-1 mouse fibroblasts (Figures 5D–5F), a classical *in vitro* model used to study *Gpx4* deficiency-induced spontaneous ferroptosis.^50^ As a control, the overexpression of *Txndc12* partly inhibited erastin-induced cell death (Figure 5G). Furthermore, the IP assay did not detect a direct interaction between TXNDC12 and GPX4 in response to erastin (Figure 5H), suggesting that TXNDC12 may not be a substrate of GPX4. As TXNDC12 is a notable antioxidant, we conducted an assay to assess its impact on Fe^2+^-induced liposome leakage. This assay relies on the de-quenching of fluorescence from entrapped dyes as they are released into the bulk solution. The assay results indicated that, like ferrostatin-1 (a radical-trapping antioxidant) and GPX4 protein, TXNDC12 protein effectively mitigated liposome damage induced by Fe^2+^ (Figure 5I).

Taken together, these findings suggest that TXNDC12 and GPX4 may play distinct roles in suppressing lipid peroxidation-induced ferroptosis, with their effects depending on the specific context.

### Targeting TXNDC12 enhances ferroptosis-mediated tumor suppression *in vivo*

**Figure 6:**
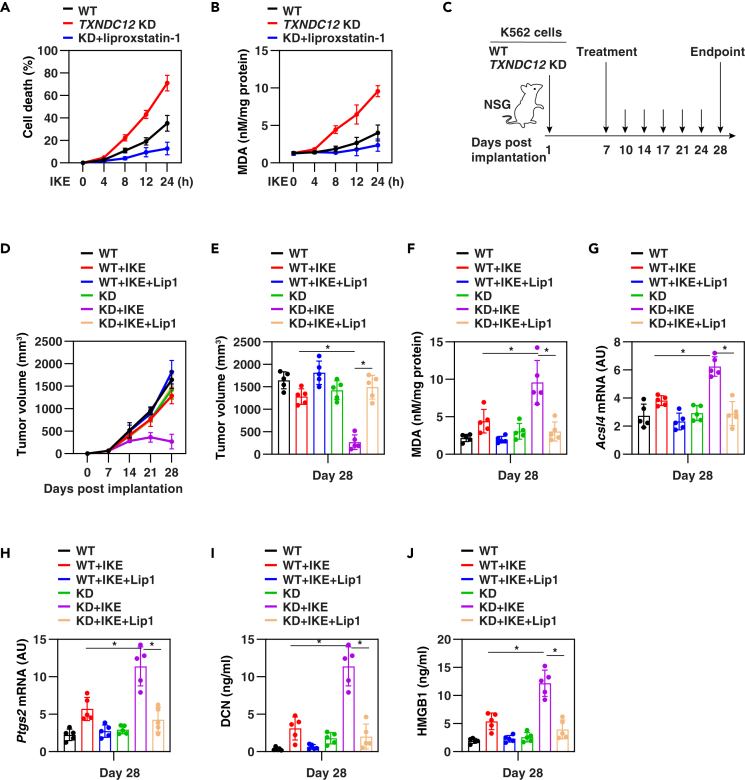
Targeting TXNDC12 enhances ferroptosis-mediated tumor suppression *in vivo* (A and B) WT and *TXNDC12*-knockdown (KD) K562 cells were treated with IKE (5 μM) for 4–24 h, and cell death (A) and intracellular MDA (B) levels were measured. The data represent the means ± SD from three independent samples. (C) Study design for the animal experiment involving NSG mice implanted with both WT and *TXNDC12*-knockdown (KD) K562 cells. (D) Tumor volume assessments (n=5 mice/group). (E–J) On day 28, tumor size (E), MDA levels (F), as well as *Acsl4* (F) and *Ptgs2* (G) gene expression in tumors, were determined, and the serum concentrations of DCN (H) and HMGB1 (J) were measured (n = 5 mice/group). Statistical analysis was performed using one-way ANOVA with Tukey’s multiple comparisons test. ∗p < 0.05.

To assess the potential of genetic inhibition of *TXNDC12* in enhancing the anticancer activity of a ferroptosis inducer *in vivo*, we utilized a selective derivative of erastin called IKE, known for its improved *in vivo* metabolic drug activity.^51^ *In vitro* studies demonstrated that K562 cells with *TXNDC12* knockdown showed an increased susceptibility to cell death and lipid peroxidation in response to IKE, compared to the control cells (Figures 6A and 6B). The inhibitory effect of liproxstatin-1 on this process (Figures 6A and 6B) suggested that knockdown of *TXNDC12* enhanced IKE-induced ferroptosis.

In a xenograft model, 1×10^7^ control and *TXNDC12*-knockdown K562 cells were implanted into immunodeficient NSG mice. Once the tumor size reached 50–80 mm^3^ at day 7, the mice were treated with vehicle or IKE (30 mg/kg, i.p.) twice weekly for three weeks (Figure 6C). Compared to the control group, the knockdown of *TXNDC12* resulted in a 3-fold increase in IKE-induced tumor suppression, which was further inhibited by the ferroptosis inhibitor liproxstatin-1 (Figures 6D and 6E). At the end of the experiment on day 28, the tumor samples were analyzed, revealing elevated levels of MDA (Figure 6F), as well as increased mRNA expression of ferroptosis biomarkers acyl-coA synthetase long chain family member 4 (*Acsl4*) (Figure 6G)^52^ and prostaglandin-endoperoxide synthase 2 (*Ptgs2*) (Figure 6H),^18^ in response to *TXNDC12* knockdown. The enzyme-linked immunosorbent assay (ELISA) assays measuring serum markers of damage-associated molecular pattern, such as decorin (DCN) (Figure 6I)^19^ and high mobility group box 1 (HMGB1) (Figure 6J),^53^ also provided support for the enhancement of ferroptosis-mediated tumor suppression *in vivo* through *TXNDC12* knockdown.

## Discussion

Ferroptosis was initially described as a form of cancer cell death dependent on *RAS* mutations, characterized by abnormal iron accumulation and oxidative stress.^9^ However, it is now known that this type of cell death can occur in normal cells or tissues under various physiological or pathological conditions.^15^^,^^54^ As an attractive therapeutic target in tumor therapy, ferroptosis faces challenges, such as drug resistance and potential toxicity effects associated with ferroptosis activators.^10^^,^^11^^,^^45^^,^^55^^,^^56^^,^^57^^,^^58^^,^^59^^,^^60^ In this study, we present an antioxidant mechanism involving ER-related TXNDC12 in human cancer cells. Moreover, we demonstrate that TXNDC12 is an ATF4-dependent target gene and provide genetic evidence that TXNDC12 inhibits lipid peroxidation and ferroptosis *in vitro* and *in vivo*. These findings contribute to our comprehension of the intricate oxidative damage and antioxidant defense pathways in ferroptosis.^61^

TXNDC12 is primarily associated with ER function and protein folding. It serves as a molecular chaperone and contributes to maintaining proper protein folding and quality control within the ER.^62^ Although the role of TXNDC12 in cancer is not extensively documented, studies suggest its potential involvement in cancer progression and cellular responses to oxidative stress. The upregulation of TXNDC12 is observed in certain cancer types, including hepatocellular carcinoma and gastric cancer, where it promotes cell growth, migration, and invasion.^43^^,^^63^ Specifically, through protein-protein interaction, TXNDC12 can activate β-catenin and facilitate the epithelial-mesenchymal transition process, thereby promoting tumor metastasis.^43^ Furthermore, high expression of TXNDC12 is correlated with poor prognosis in patients with glioma and lung cancers, indicating its potential broader role in cancer development and progression.^64^^,^^65^^,^^66^ In the present study, we reveal that TXNDC12 exhibits selective upregulation specifically during ferroptosis, while no significant changes are observed during apoptosis in leukemia cells. Functionally, we demonstrate that targeting TXNDC12 can effectively enhance the sensitivity of cancer cells to erastin or RSL3-induced ferroptosis.

ATF4 is a transcription factor that plays a crucial role in the cellular response to ER stress.^67^ ER stress occurs when there is an imbalance between protein folding demand and capacity in the ER, leading to the accumulation of misfolded or unfolded proteins. ATF4 is activated in response to ER stress and serves as a central regulator of the unfolded protein response, a cellular stress response pathway. Its main function during ER stress is to promote adaptive responses that restore ER homeostasis and alleviate stress.^67^ ATF4 induces the transcription of various target genes involved in protein folding, ER-associated degradation, amino acid metabolism, antioxidant defense, and autophagy.^68^^,^^69^^,^^70^^,^^71^ We demonstrate that TXNDC12 is a ATF4-targeted gene involved in driving ferroptosis resistance. While we demonstrate that NFE2L2 is not required for inducible TXNDC12 expression, the activation of NFE2L2 may upregulate other antioxidant or damage protection genes (such as NAD(P)H quinone dehydrogenase 1 [NQO1], ferritin heavy chain-1 [FTH1], and metallothionein 1G [MT1G]) involved in the prevention of ferroptosis.^34^^,^^37^^,^^72^^,^^73^^,^^74^ Hence, the activation of distinct transcription mechanisms may be crucial for uncovering downstream genes and pathways involved in oxidative damage.

Our study highlights that TXNDC12 inhibits lipid peroxidation and ferroptosis in a GPX4-independent manner. TXNDC12, as a disulfide-containing protein, exhibits antioxidant activity. The upregulation of TXNDC12 expression serves as a defense mechanism against lipid oxidation-induced toxicity. GPX4 is primarily known for its function in preventing lipid peroxidation, a process where reactive oxygen species attack and damage lipids in cell membranes.^75^ While GPX4 is expressed in the cytosol, mitochondria, and nucleus, cytosolic GPX4 appears to play a major role in inhibiting ferroptosis.^18^ In addition, GPX4 also inhibits non-ferroptotic damage under certain conditions.^22^ Given that TXNDC12 is predominantly expressed in the ER, different organelles possess distinct defense mechanisms to mitigate membrane damage caused by lipid peroxidation. Recent studies indicate that erastin and RSL3 have multiple targets beyond system xc^−^ and GPX4.^76^^,^^77^ Our study indicates that TXNDC12 possesses a similar ability to GPX4 in preventing iron-induced lipid peroxidation, as demonstrated through liposome leakage experiments. A comprehensive understanding of the defense mechanisms and membrane repair processes against organelle damage in ferroptosis is essential for the advancement of innovative anticancer strategies.^78^^,^^79^^,^^80^

In summary, TXNDC12 plays a pivotal role in inhibiting lipid peroxidation in leukemia cells in both *in vitro* and *in vivo* settings. Targeting this TXNDC12-dependent resistance mechanism could enhance ferroptosis-induced tumor suppression.

### Limitations of the study

While our study suggests that TXNDC12 and GPX4 play distinct roles in inhibiting lipid peroxidation, further investigation is needed to understand the location-dependent functions and relationship between TXNDC12 and GPX4 in ferroptosis.^81^ Although the immunoprecipitation experiments indicate that TXNDC12 and GPX4 do not directly interact, it is imperative to utilize a cell-free system to definitively determine whether TXNDC12 functions as a substrate of GPX4. Nonetheless, defining both GPX4-dependent and independent pathways in ferroptosis remains critical for the development of effective anticancer strategies.

## STAR★Methods

### Key resources table

**Table:** 

REAGENT or RESOURCE	SOURCE	IDENTIFIER
**Antibodies**		

TXNDC12	Thermo Fisher Scientific	PA5-120272; RRID:AB_2913844
TXNDC5	Thermo Fisher Scientific	PA5-83241; RRID:AB_2790397
HSPA5	Cell Signaling Technology	3177; RRID:AB_2119845
C-CASP3	Cell Signaling Technology	9664, RRID:AB_2070042
C-PARP1	Cell Signaling Technology	5625; RRID:AB_10699459
ATF4	Cell Signaling Technology	11815; RRID:AB_2616025
NFE2L2	Cell Signaling Technology	20733; RRID:AB_2934224
SLC7A11	Cell Signaling Technology	12691; RRID:AB_2687474
AIFM2	Cell Signaling Technology	24972; RRID:N/A
DHODH	Cell Signaling Technology	26381; RRID:N/A
ACTB	Cell Signaling Technology	3700; RRID:AB_2242334
GPX4	Abcam	ab125066; RRID:AB_10973901

**Chemicals, peptides, and recombinant proteins**		

Erastin	Selleck Chemicals	S7242
RSL3	Selleck Chemicals	S8155
Staurosporine	Selleck Chemicals	S1421
Liproxstatin-1	Selleck Chemicals	S7699
Ferrostatin-1	Selleck Chemicals	S7243
Z-VAD-FMK	Selleck Chemicals	S7023
ISRIB	Selleck Chemicals	S0706
ML385	Selleck Chemicals	S8790
Buthionine sulphoximine	Selleck Chemicals	S9728
Lipofectamine 3000	Invitrogen	L3000-015
Puromycin	InvivoGen	ant-pr-1
SuperSignal west pico chemiluminescent substrate	Thermo Fisher Scientific	34580
SuperSignal west femto maximum sensitivity substrate	Thermo Fisher Scientific	34095
Cell lysis buffer	Cell Signaling Technology	9803
PrimeScript RT Master Mix	Takara	RR037A
SsoFast EvaGreen Supermix	Bio-Rad	172-5204
rGPX4	OriGene	TP762179
rTXNDC12	OriGene	TP303511

**Critical commercial assays**		

BCA Protein Assay Kit	Thermo Fisher Scientific	23225
Cell Counting Kit-8	Sigma	96992
Neon Transfection System Kit	Thermo Fisher Scientific	MPK10096
RNeasy Plus Micro Kit	QIAGEN	4034
Dual-Luciferase® Reporter Assay System	QIAGEN	N1610
Iron Assay Kit	Abcam	ab83366
MDA Assay Kit	Abcam	ab233471
Protein A magnetic beads	MilliporeSigma	LSKMAGA10
Q5® Site-Directed Mutagenesis Kit	New England Biolabs	E0554S
SimpleChIP Enzymatic Chromatin IP Kit	Cell Signaling Technology	9003
QIAquick PCR Purification Kit	QIAGEN	28104
HMGB1 ELISA Kit	Shino Test Corporation	326054329
DCN ELISA Kit	Thermo Fisher Scientific	EMDCN

**Experimental models: Cell lines**		

HL60	ATCC	CCL-240
K562	ATCC	CCL-243
MCF7	ATCC	HTB-22
U2OS	ATCC	HTB-99
PANC1	ATCC	CRL-1469
HCT116	ATCC	CCL-247
SW48	ATCC	CCL-231
293FT cells	Thermo Fisher Scientific	R70007

**Experimental models: Organisms/strains**		

NOD mice	The Jackson Laboratory	032455

**Oligonucleotides**		

Human *TXNDC1* primers: GAAGACCTTGGATTGCCAGTGTG and GAAGGACAAAGGCAATCTGCCAC	Sigma-Aldrich	N/A
Human *TXNDC2* primers: CTCAGGAAGAAACAAGTGAAGGTG and TGTGGAACGTGTGGCTGACCAT	Sigma-Aldrich	N/A
Human *TXNDC3* primers: GTCGAATAGCAGACCAGTGTGAC and CGTTAGGTTCGGTGTCAGTCTG	Sigma-Aldrich	N/A
Human *TXNDC4* primers: AGTAGTGTTTGCCAGAGTTGATTG and CTGCCAATGCTTTCACTGATCGC	Sigma-Aldrich	N/A
Human *TXNDC5* primers: GTAGACTGCACTGCTGAACGGA and TCGTCTTTCGCTTGGCTCAGGA	Sigma-Aldrich	N/A
Human *TXNDC6* primers: TCACTACCTGGCGAACCGTCAT and GCAATGCCAGTTCTCTGTCAGC	Sigma-Aldrich	N/A
Human *TXNDC7* primers: TCAGAAAGGCGAGTCTCCTGTG and CCTCTTGGCAATGTCCTCGTTG	Sigma-Aldrich	N/A
Human *TXNDC8* primers: GAAACGGTGTGGTCCCTGCAAA and TGATGTGACAAGTTTCAGCCAGC	Sigma-Aldrich	N/A
Human *TXNDC9* primers: GACTCCAGGCACTAAGGAAAGC and GGCAAACCACATTTTCACTCTCC	Sigma-Aldrich	N/A
Human *TXNDC10* primers: CATTTTGGATGGCACAGTAGAAGC and GAGAAAGCAGCCCATCAGTGGT	Sigma-Aldrich	N/A
Human *TXNDC11* primers: GCATCTCATTGGAAGTGGCTCTG and ACGGAGCGTAATAGAGCAGGAG	Sigma-Aldrich	N/A
Human *TXNDC12* primers: GGACATAATGGGCTTGGAAAGGG and CTTTGCAAGCTCCACACCAGGA	Sigma-Aldrich	N/A
Human *TXNDC13* primers: CCATCCTGCCAGCAGACTGATT and GAGAGTGGTGACAAAGAAGCGG	Sigma-Aldrich	N/A
Human *TXNDC14* primers: TTGGGAAGGTGGATGTTGGACG and CTTGCCACCTTGGAACAGGATC	Sigma-Aldrich	N/A
Human *TXNDC15* primers: GCCGCTTTTCTGCCAGTTTGGC and GAACAGCTACGGTGCCAAACCT	Sigma-Aldrich	N/A
Human *TXNDC16* primers: CCATCTCCAACTGGGCTTACCA and CTGCTTTTCCCAGAAGACGCCA	Sigma-Aldrich	N/A
Human *TXNDC17* primers: CGCCTACTTTACGGGTTCTAAGG and GGCTTTTCTCCTACTTGGCAGTA	Sigma-Aldrich	N/A
Human *TXNDC1* primers: 5'-TGGCAGTGAGAGAAAACCGCAC-3' and 5'-CTAAGCTGGTGAGGCTCCTGTT-3'	Sigma-Aldrich	N/A
*Human ATF4*-shRNA: GCCTAGGTCTCTTAGATGATT	Sigma-Aldrich	N/A
Human *NEF2L2*-shRNA: CCGGCATTTCACTAAACACAA	Sigma-Aldrich	N/A
Human *TXNDC12*-shRNA: CCTGATGGTGATTATTCATAA	Sigma-Aldrich	N/A
Human *TXNDC12*-gRNA: AAGATTTCAGCCCTGACGG	Sigma-Aldrich	N/A
Human or mouse *TXNDC12* cDNA	OriGene	MC200299 and SC114591
Human *GPX4* cDNA	OriGene	RC208065

**Software and algorithms**		

Image Lab software	Bio-Rad	http://www.bio-rad.com/en-us/sku/1709690-image-lab-software
GraphPad Prism 9.0	GraphPad Software	http://www.graphpad.com/

### Resource availability

#### Lead contact

Further information and requests for resources and reagents should be directed to and will be fulfilled by the lead contact, Qingnan He (email: heqn2629@163.com).

#### Materials availability

The plasmids and cell lines generated in this study are available from the lead contact upon request.

#### Data and code availability

• Data reported in this paper will be shared by the lead contact upon request.
• This paper does not report original code.
• Any additional information required to reanalyze the data reported in this paper is available from the lead contact upon request.

### Experimental model and study participant details

#### Mice

All animal experiments were conducted in accordance with the guidelines of the Association for Assessment and Accreditation of Laboratory Animal Care (http://www.aaalac.org) and approved by the institutional animal care and use committees (Central South University [XMXH-2023-0101] and Guangzhou Medical University [2023021]). Mice were housed in ventilated animal cabinets under controlled lighting conditions (12/12 h light-dark cycle), at a temperature of 20-25°C and 65% humidity, in a super pathogen-free environment. They had *ad libitum* access to food and water. Experiments were carried out under pathogen-free conditions, and the health status of mouse lines was routinely checked by veterinary staff. Euthanasia of the animals was performed by exposing them to CO_2_.

K562 (female) cells were maintained in suspension culture at the exponential phase of growth, with a minimum of 98% cell viability. Male and female NOD mice (NOD/ShiLtJ strain), in a 1:1 ratio, aged between 10 and 12 weeks, were utilized for the experiments. Each mouse received a single subcutaneous injection of 1×10^7^ K562 cells suspended in 100 microliters of PBS into the flank of a hind leg. On day 7, when the tumor volume reached 50-80 mm^3^, the mice were randomly assigned to the respective treatment groups. The mice were treated twice weekly with either the vehicle or IKE (30 mg/kg, i.p.) in the absence or presence of liproxstatin-1 (10 mg/kg, i.p.) for a duration of three weeks. Tumor volumes were measured weekly, and their values were calculated using the formula: length × width^2^ × π/6. Tumor and blood samples were collected on day 28 after implantation for further assays.

#### Cell lines

The human cancer cell lines HL60 (female; CCL-240), K562 (female; CCL-243), MCF7 (female; HTB-22), U2OS (female; HTB-99), PANC1 (male; CRL-1469), HCT116 (male; CCL-247), and SW48 (female; CCL-231) were obtained from the American Type Culture Collection. *Gpx4*-knockout Pfa1 cells (male) were generously provided by Dr. Marcus Conrad. ATF4-inducible 293T cells (male) were purchased from YunZhou Biology. These cells were cultured in RPMI-1640 Medium or Dulbecco's modified eagle's medium supplemented with 10% fetal bovine serum, 2 mM l-glutamine, and 100 U/ml penicillin and streptomycin. The cells were maintained in a humidified incubator at 37°C with 95% humidity and 5% CO_2_.

All cell lines used were authenticated using short tandem repeat profiling, and mycoplasma testing was negative. When utilizing dimethyl sulfoxide (DMSO) as a drug lysis reagent, the final concentration of DMSO in the working solution was maintained below 0.01%. A 0.01% DMSO concentration was used as a carrier control in the corresponding cell assays.

### Method details

#### Western blot analysis

After stimulation, cells were washed with phosphate-buffered saline (PBS) and lysed on ice for 10 minutes using cell lysis buffer (Cell Signaling Technology, 9803) supplemented with a protease/phosphatase inhibitor cocktail (Cell Signaling Technology, 5872).^82^^,^^83^ The lysate was then centrifuged at 14,000g for 10 minutes at 4°C to remove cell debris. Protein concentrations were determined using a bicinchoninic acid (BCA) protein assay kit (Thermo Fisher Scientific, 23225). Subsequently, 25 μg of protein from each sample was loaded onto 4%-12% Criterion XT Bis-Tris gels in XT MES running buffer and transferred to polyvinylidene difluoride membranes using the Trans-Blot Turbo Transfer Pack and System (Bio-Rad). The polyvinylidene difluoride membranes were blocked with 5% skim milk for 1 hour, followed by overnight incubation at 4°C with primary antibodies (1:1000-1:2000 dilution). After incubation with peroxidase-conjugated secondary antibodies (1:1000) for 1 hour at room temperature, the membranes were washed five times for 5 minutes each with TBST (Tris-buffered saline, 0.1% Tween 20). The signals were visualized using enhanced chemiluminescence, and the blots were analyzed using the ChemiDoc Touch Imaging System (Bio-Rad).

#### qPCR analysis

Total RNA was extracted using the RNeasy Plus Micro Kit (QIAGEN, 74034) following the manufacturer's instructions. Cells were lysed and homogenized in a denaturing Buffer RLT Plus containing guanidine isothiocyanate. The cell lysate was then passed through a gDNA Eliminator column to remove double-stranded DNA. Total RNA was purified using a RNeasy MinElute centrifuge column. First-strand cDNA was synthesized from 1 μg of RNA using the PrimeScript RT Master Mix (Takara, RR037A). The synthesized cDNA was subjected to qPCR reaction. Each 10 μl reaction contained 5 μl of SYBR Green qPCR Master Mix, 4 μl of diluted cDNA, and 0.5 μl of each forward and reverse primer (10 μM). The qPCR was performed using SsoFast EvaGreen Supermix (Bio-Rad, 172-5204) on the C1000 Touch Thermocycler CFX96 Real-Time System (Bio-Rad). The data were normalized to RNA GAPDH, and the fold change was calculated using the 2^-DDCt^ method.^84^ The relative concentrations of mRNA were expressed in arbitrary units, with the untreated group assigned a value of 1.

#### RNAi, gene editing, and gene transfection

Predesigned human *ATF4*-shRNA (GCCTAGGTCTCTTAGATGATT), human *NEF2L2*-shRNA (CCGGCATTTCACTAAACACAA), human *TXNDC12*-shRNA (CCTGATGGTGATTATTCATAA), and control shRNA were purchased from Sigma-Aldrich. Human or mouse *TXNDC12* cDNA (MC200299 and SC114591) and human *GPX4* cDNA (RC208065) were obtained from OriGene. The cDNA for *TXNDC12-CS* was generated as previously described.^43^ The Invitrogen Neon Transfection System (Thermo Fisher Scientific) was utilized to deliver shRNA or cDNA into leukemia cells using the following conditions: 1,400 V/10 ms/3 pulses in 24-well plates, following the manufacturer's instructions. Stable cell lines were further selected through antibiotic screening. CRISPR-Cas9–mediated gene editing was performed in close adherence to Feng Zhang lab’s protocol.^85^ *TXNDC12*-gRNA (sequence: AAGATTTCAGCCCTGACGG) was predesigned and purchased from Sigma-Aldrich.

#### Cell viability and death assay

The cell viability was assessed using a Cell Counting Kit-8 (CCK-8) kit (Sigma-Aldrich, 96992) following the manufacturer's protocol. Leukemia cell lines were seeded in 96-well plates at a density of 1×10^4^ cells/well and treated with the indicated compounds for the specified durations (4-24 hours). After treatment, a 10 μL CCK-8 working solution was added to the medium, and the plates were incubated at 37°C for 60 minutes. The absorbance at 450 nm was measured using a microplate reader, with the absorbance value being proportional to the number of viable cells in the culture. For cell death assessment, the Countess 3 Automated Cell Counter (Thermo Fisher Scientific Inc) was utilized. Cells were stained with trypan blue, and the percentage of cell death was determined by counting the stained (non-viable) cells.

#### Iron assay

The relative level of free ferrous iron (Fe^2+^) in cells was determined using an iron assay kit (Abcam, ab83366) following the manufacturer's instructions. The kit provided the iron standard and all necessary buffers. The assay involved the following main steps: setting up reaction wells; adding 5 μL of iron reducer to each standard well; adding 5 μL of assay buffer to each sample; mixing and incubating the standards and samples at 37°C for 30 minutes; adding 100 μL of iron probe to each well containing the iron standard and test samples; mixing and incubating them at 37°C for 60 minutes, protected from light. The output was measured immediately using a colorimetric microplate reader at OD 593 nm.

#### MDA assay

The concentration of MDA in cells or tissues was determined using an MDA assay kit (Abcam, ab233471) following the manufacturer's instructions.^86^^,^^87^ The kit provided MDA standard and all necessary buffers. The assay involved the following main steps: serial dilution of MDA standards and test samples; addition of 10 μl of MDA color reagent stock solution to each well of MDA standard; incubation at room temperature for 10-30 minutes; addition of 40 μl of reaction solution and further incubation at room temperature for 30-60 minutes; measurement of the end-point absorbance at OD 695-700 nm using an absorbance plate reader with path-check correction.

#### ChIP assay

We performed ChIP using the SimpleChIP Enzymatic Chromatin IP Kit (Cell Signaling Technology) following the manufacturer's instructions. In brief, we cross-linked 1 × 10^7^ K562 cells with 1% fresh formaldehyde and incubated them for 10 minutes at room temperature. Subsequently, we lysed the cells and enzymatically digested chromatin to obtain DNA fragments ranging from 150 to 900 base pairs in length. The chromatin fragments were then subjected to immunoprecipitation using anti-ATF4 antibodies (Cell Signaling Technology; 11815; diluted 1:50) at 4°C overnight with rotation. Following this, we incubated the chromatin-antibody complexes with protein G magnetic beads at 4°C for 2 hours. After eluting the chromatin from the antibodies and reversing the formaldehyde-induced cross-linking, we purified the DNA using the QIAquick PCR Purification Kit (QIAGEN). Finally, we analyzed both the immunoprecipitated DNA and input DNA through qPCR.

#### Luciferase assay

We cultured 293FT cells (Thermo Fisher Scientific) at a density of 5 × 10^4^ cells per well in 24-well plates. Each well received transfection with 0.05 μg of Renilla luciferase plasmids (Addgene) and 0.5 μg of the pGL3 basic luciferase vector containing either the WT or mutated human *TXNDC12* promoter.^88^ Additionally, we included 1 μg of vector or pMIEG3-ATF4 plasmids in the transfection mixture. Lipofectamine 3000 reagent (Invitrogen) was employed for the transfection process. After a 24-hour incubation post-transfection, we assessed luciferase activity using the Dual-Luciferase Reporter Assay System (Promega) in a luminometer. To ensure accuracy, we normalized the firefly luciferase activity with the corresponding Renilla luciferase activity.

#### Liposome leakage assay

Liposomes were prepared by a filming-rehydration method.^89^ The phospholipids used in this work were all obtained from Avanti and were dissolved in chloroform and then subjected to a rotary evaporator to remove the solvent in flask, thus leading to lipid film formation. The neutral-pH hydration buffer was composed of 100 mM KCl, 10 mM Tris, 100 mM MES, pH 7.0. The fluorescent dye calcein was dissolved in the hydration buffer to make a saturated solution. The liposomes encapsulating calcein were prepared by adding the saturated solution of calcein to the flask with lipid film, followed by treatment in an ultrasonic water bath. The thus-formed liposomes were extruded through 0.1 μm nucleopore polycarbonate membranes by using an Avanti Mini-Extruder (Avanti Polar Lipids, Inc). The free calcein was removed by gel filtration on a Sephadex G-50 column (GE Healthcare, 17004201), and the liposomes were collected and adjusted to a lipid concentration of 0.8 mg/mL before use. The liposomes were incubated with Fe^2+^ (10 μM) under conditions either with or without the presence of ferrostain-1 (1 μM), rGPX4 (200 nM; TP762179, OriGene), or rTXNDC12 (200 nM; TP303511, OriGene) for a duration of 20 minutes. The liposome leakage was measured by monitoring the fluorescence intensity at preset time points with an excitation wavelength of 490 nm and emission wavelength of 520 nm using a microplate reader.

#### IP assay

Cell lysis was performed at 4°C using ice-cold IP buffer composed of 2% sodium dodecyl sulfate (SDS), 10 mM Tris-HCl (pH 8.0), and 150 mM NaCl, supplemented with a protease inhibitor cocktail.^13^^,^^90^ The cell lysates were then clarified by a brief centrifugation step (13,000g, 15 minutes). Protein concentrations in the resulting supernatants were determined using the BCA assay (Thermo Fisher Scientific, 23225). Prior to immunoprecipitation, equal amounts of protein from each sample were pre-cleared by incubation with protein A agarose beads (4°C, 3 hours; Santa Cruz Biotechnology, sc-2027). Subsequently, the pre-cleared samples were incubated with either irrelevant IgG or specific antibodies (3 μg/ml) in the presence of protein A agarose beads for 2 hours or overnight at 4°C with gentle shaking. After the incubation period, agarose beads were thoroughly washed with PBS, and the proteins were eluted by boiling them in 2× SDS sample buffer before performing SDS–polyacrylamide gel electrophoresis.

#### ELISA assay of HMGB1 and DCN

The quantitative determination of damage-associated molecular patterns in mice serum was performed using commercially available HMGB1 (Shino Test Corporation, 326054329) and DCN (Thermo Fisher Scientific, EMDCN) ELISA kits. The samples were prepared and assayed following the protocols provided by the respective manufacturers. The ELISA assays were carried out with appropriate dilutions of the serum samples and standard controls provided by the kits, and the absorbance was measured using a microplate reader as specified by the manufacturers' protocols. Data analysis was performed according to the standard curve generated from the known concentrations of the standards.

### Quantification and statistical analysis

Data collection and statistical analysis were performed using GraphPad Prism 9 software. Comparisons among the different groups were conducted using a one-way or two-way analysis of variance (ANOVA), followed by Tukey's multiple comparisons test. The results were presented as mean ± standard deviation (SD) of three independent experiments except where otherwise indicated. Statistical significance was determined using a significance level of P < 0.05. No methods were used to determine whether the data met assumptions of the statistical approach. Statistical details of the experiments can be found in the figure legends. The exact value of n within the figures is also indicated in the figure legends.
